# SMAD4 mutations identified in Iranian patients with colorectal cancer and polyp 

**Published:** 2021

**Authors:** Rouhallah Najjar Sadeghi, Nastaran saeedi, Negar sahba, Amir Sadeghi

**Affiliations:** 1 *Basic and Molecular Epidemiology of Gastrointestinal Disorders, Research Institute for Gastroenterology and Liver Diseases, Shahid Beheshti University of Medical Sciences, Tehran, Iran*; 2 *Faculty of Medicine, Department of Clinical Biochemistry, Mazandaran University of Medical Sciences, Sari, Iran *; 3 *Gastroenterology and Liver Diseases Research Center, Research Institute for Gastroenterology and Liver Diseases, Shahid Beheshti University of Medical Sciences, Tehran, Iran*

**Keywords:** SMAD4, MADH4, DPC4, Colorectal cancer, MH2 domain, Neoplastic polyp

## Abstract

**Aim:**

Search for SMAD4 mutations in Colorectal cancer (CRC) or polyp in Iran.

**Background:**

Colorectal cancer is one of the five prevalent cancers among the Iranian population; however, its molecular mechanisms are not fully understood. The vast majority of CRCs arise from neoplastic polyp

**Methods:**

Colorectal cancer and polyp lesions with matched normal tissues from patients who had undergone colonoscopy in Taleghani Hospital (January 2009 – November 2010) were included in the study. DNA extraction and PCR-sequencing for exons 5-11 of the SMAD-4 gene were carried out on 39 and 30 specimens of polyp and adenocarcinoma, respectively.

**Results:**

Of cancer and polyp specimens, 33.3% and 28.2%, respectively, were mutated in the Smad-4 gene. The majority of SMAD4 mutations, especially in the MH_2_ domain were missense mutations (63.6% and 68.75, respectively). In cancer, codon 435 and in polyp, codons 435 and 399 were the most common alterations. Unlike cancer specimens, transversion was found frequently in the polyp (56.25% vs. 35.7%). CG>TA transition was about 18.75% and 14.3% in cancer and polyp samples, respectively. Mutations of codon 264 and C.483-4 were seen both in cancer and neoplastic polyps.

**Conclusion:**

As frequent alterations, missense mutations are presumably selected during tumorigenesis and polyposis due to their structural impacts on SMAD4 functions and TGF-ß signaling pathway. The lower frequency of CG>TA can be attributed to global genome hypomethylation. Presumably, SMAD4 mutations had occurred in the primary polyps, and some of these mutated cells then developed into carcinoma. On the other hand, polyp-specific mutations may lower the risk of CRC.

## Introduction

 The first specified substrate of TGF-β receptor kinases is the proteins of the SMAD family (1). Through their phosphorylation and activation by transmembrane receptor with serine-threonine kinase activity, members of the SMAD family play decisive roles in cell functions (2). 

As an essential effector in the TGF-β pathway, SMAD4 acts as a mediator of extracellular growth factors inside the cell nucleus (3). SMAD4 is known to regulate cell proliferation (4), differentiation (5), and apoptosis (6), and upon loss of SMAD4 expression, the cell growth and apoptosis are no longer inhibited by TGF-β (7).

SMAD4 contains conserved MH_1_ and MH_2_ domains in the C- and N-terminals, respectively, which are separated by a linker domain rich in proline. The MH_1_ domain has an intrinsic DNA binding activity, while the MH_2_ domain involves the biological effects such as interaction with regulatory proteins. Moreover, through intramolecular interaction, the MH1 domain suppresses the biological and transcriptional activities of the MH2 domain (8). 

**Table 1:** List of primers and cycling program of p53 gene (exons 5, 6 and 8-11).

Region (PCR Product)	Table 1	Sequence (5'>3')	Cycle*	[MgCl_2_]
Exon 5&6 (845 bp)	Forward	CTGATAGGCCATGGGTGAGT	94ºC(35 s), 63.2ºC (40 s),72ºC (45 s)	1.5mM
	Reverse	CTACGCTGAGGGAAACCTTG		
Exon 8 (676 bp)	Forward	GTTGACCTGGTCCTTTGAG	94ºC ( 35s), 55.4ºC (40 s),72ºC (45 s)	1.2 mM
	Reverse	CCGACAATTAAGATGGAGTG		
Exon 9 (483 bp)	Forward	TCATACTACATGCTCCTGACAC	94ºC ( 30s), 59.8ºC (30 s), 72ºC ( 45 s)	1.4mM
	Reverse	TTTCCATTCCTTCCACCCAG		
Exon 10 (580 bp)	Forward	GACATGATCTTCTTGGTGAGC	94ºC ( 30s), 58.2ºC (35 s), 72ºC ( 40 s)	1.4mM
	Reverse	ATCCCCTTTCTCCTTCATCC		
Exon 11 (650 bp)	Forward	ACTTCTTGGCACTTTAGCAGAG	94ºC (35 s), 52.9ºC (35 s), 72ºC (45 s)	1.2 mM
	Reverse	GGGCTAAATTTTCTAGCACTGG		

*Considering that for all reactions, the initial denaturation and final extension were 5 minutes at 94^o^C and 72 ^o^C for 10 minutes respectively

The majority of SMAD4 alterations cluster in the MH_2_ domain and often alter residues in the vicinity of protein interface mediating SMAD4 hetero-oligomerization (9). Mutations at the MH_1_ domain have been reported to enhance interactions with the MH_2_ domain (10) and alter DNA binding (11), protein stability, and prevent nuclear translocation (12).

SMAD4 is the subject of inactivating mutations in some cancers, and loss of SMAD4 expression is a notable feature of most human cancers (3), including colorectal cancer (CRC) (13). SMAD4 mutations were reported in 2.1–31% of colorectal cancer cases (14-23).

Nevertheless, previous studies have established some associations between SMAD4 mutation and protein expression with the survival of patients and progress of colorectal cancer (24).

CRC is the second and third most common and lethal cancer in males and females, respectively, worldwide, and more than 1.8 million new cases and 881,000 deaths were estimated to have occurred in 2018 (25). Among Iranian males and females, CRC is one of the five most common cancers (26, 27), accounting for approximately 6.3% of all cancer deaths; 3641 new cases and 2262 deaths from CRC are estimated annually (28). While the incidence rate of colorectal decreased annually in the USA during 1975-2017 (29), the rate is rapidly increasing in several regions historically at low risk (30) and in younger generations of Iran (31).

There are several pathways for CRC (32). The vast majority of CRCs arise from precursor lesions, termed polyps (33), and the adenoma-carcinoma sequence accounts for nearly 95% of all CRCs (34). Moreover, 15-20% of sporadic CRC develops from serrated polyps through pathways distinct from the traditional adenoma-carcinoma sequence (35). 

Given the earlier studies on the importance of SMAD4 integrity and considering the prevalence of CRC in Iran, this study was designed to evaluate the contribution of SMAD4 mutations in colorectal carcinogenesis and polyposis and their correlation with clinicopathological aspects. To date, no attempt has been made to search for SMAD4 mutations in CRC or polyp in Iran. 

## Methods

**Patients**

Colorectal cancer (intestinal-type) and polyp lesions with matched normal tissues were collected from patients who had undergone a colonoscopy of the gastrointestinal tract in Taleghani Hospital (January 2009 – November 2010, Tehran, Iran). After resection, the specimens were immediately processed for the DNA extraction or were frozen at -80 °C until extraction. Specimens were obtained under informed consent and the patients were considered competent to decide to enroll. This study was approved by the Ethics and Scientific Committee of our institution following the principles of the Declaration of Helsinki. The samples were histologically diagnosed by pathologists as being CRC and polyp; only samples containing at least 80% tumor nuclei were selected for DNA extraction. 

**DNA extraction**

DNA from cancer, polyp, and normal adjacent specimens was extracted using QIAamp DNA Mini Kit (Qiagen, Hilden, Germany) according to the manufacturer’s instructions. 

**Sequence analysis for mutations detection**

In search of nucleotide alterations of the SMAD4 gene in exons 5, 6, and 8-11, PCR sequencing was carried out using primers as presented in Table 1. Primers were designed based on GenBank sequence NG_013013.2 (GI: 383387807). PCR reactions containing 10 pmol of each primer, 200 mM of each dNTP, and 0.5 U Taq polymerase were conducted in the cycling program, as shown in Table 1. Considering that for all reactions, the initial denaturation and final extension were 5 minutes at 94 ℃ and 72 ℃ for 10 minutes, respectively. DNA sequencing was performed using the ABI3130X Genetic Analyzer. To distinguish somatic mutations from germline mutations, the mutant sequences from tumor/polyp were compared with the sequence of DNA extracted from blood leucocytes of the same person. 

**Statistical analysis**

SPSS 20.0 was used for statistical analyses. The association of SMAD4 nucleotide alterations and clinical parameters, such as location and histological type of polyps, was evaluated using the Fisher exact test. A *p*-value< 0.05 was regarded as statistically significant. 

**Table 2:** Demographic information, specimen’s location and types of polyps

			Cancer	Polyp	Type of polyp		
Age (year± SD)			54.6 ± 15.9	54.1 ± 16.3			
Gender (number)	Male		13	26	Neoplastic polyps (number)	Tubular adenomas	15
	Female		17	13		Tubulovillous	6
Location (number)	Colon	Ascending	1	5		villous	3
		Cecum	1	5		Serrated polyps	2
		Descending	1	5		Juvenile polyps	3
		Hepatic flexure	3	2	Non-neoplastic polyps (number)	Hyperplastic polyps	8
		Sigmoid	3	9		Inflammatory polyps	2
		Transverse	1	3			
		Splenic flexure	0	3			
	Rectum		20	7			

## Results

**Patient characteristics**

In this study, 39 and 30 fresh tissue specimens for colorectal polyp and intestinal-type adenocarcinoma, respectively, and adjacent normal tissue were examined for the desired sequences of the SMAD4 gene. The characteristics of patients are given in Table 2.

Colorectal polyps are classified histologically as neoplastic or non-neoplastic (Table 2). The majority of samples were tubular adenomas (38.5%), hyperplastic polyps (20.5%), and tubulovillous adenomas (15.4%). 

**The spectrum of somatic mutations of SMAD4**

Overall, 33.3% (10/30) of intestinal-type adenocarcinomas and 28.2% (11/39) polyp specimens had 1-4 mutations. In cancer samples, two specimens harbored three mutations, and in polyps, two and one sample harbored two and four mutations, respectively (Table 3).

In cancer tissues, 14 mutations were detected in the coding regions and intronic region of the SMAD4 gene. In the coding regions, most of the mutations clustered in the MH_2_ domain (7 missense mutations out of 11 = 63.6%), and the remainder (36.4%) were mapped to the linker region including two missense and two silent mutations. Overall, nine missense (64.3%), two silent (14.3%), and three intronic (21.4%) mutations were identified in cancer. Missense mutations at codon 435 (ATA>GTA) were the most frequent mutation (5/14, 35.7%) in cancer patients (Figure 1A). 

In the search for SMAD4 aberrations in polyp samples, 16 mutations were detected: 11 missense (68.75%) in the MH2 region, two nonsense mutations (12.5%), and one missense (6.25%) in the linker region (3/16, 18.75%); two intronic mutations (12.5%) were also detected. Patients with polyp mostly showed mutations at codons 435 and 399 (each 25%) (Figure 1B and Table 3). 

**Type of mutations**

**Table 3:** Mutant specimens of polyp and cancer tissues

Exon Codon	Exon 5 242	Exon 6 264	Exon 6 271	Exon 8 361	Exon 9 386	Exon 9 399	Exon 9 435	Exon 10 465	c.262+80^§^	c.482+66	c.483-4
Type of mutation	Nonsense	Missense	Silent	Missense	Missense	Missense	Missense	Missense			
Mutation	TCA>TGA	AGC>AGG	AGT>AGC	CGC>CAC	GGT>GAT	GTC>CTC	ATA>GTA	GTG>ATG	TGT > G	CAG > T	TGT> A
Amino acid	Ser > Stop	Ser > Arg	Ser > Ser	Arg > His	Gly > Asp	Val > Leu	Ile > Val	Val > Met			
Region	Linker	Linker	Linker	MH_2_	MH_2_	MH_2_	MH_2_	MH_2_	Intron	Intron	Intron
8P^*^	CG										
19P						GC					
39P						GC					
42P	CG					GC					
58P							AG				
66P											TA
67P							AG				
68P		CG					AG				
77P				GA	GA	GC	AG				
86P										GT	
104P					GA						
15C^*^							AG				
26C							AG				
40C								GA			
64C							AG				
79C							AG				
81C		CG	TC						TG		
82C		CG	TC						TG		
92C					GA						
97C							AG				
122C											TA

* P: Polyp, C: Cancer. § C: Codon

**Table 4:** Different distribution of mutations in polyp and cancer samples from view of transition and transversion

		Codon(s)	Polyp *	Cancer*
Transition	CG > TA	465, 361, 386	3 (18.75)	2 (14.3)
	TA > CG	271, 435	4 (25)	7 (50)
Transversion	CG > AT	C.482+ 66	1 (6.25)	0 (0)
	CG > GC	242, 264, 399	7 (43.75)	2 (14.3)
	TA > AT	C.484- 4	1 (6.25)	1 (7.1)
	TA > GC	C.262+ 80	0 (0)	2 (14.3)

* Number (%)

**Table 5:** The frequency of mutations in each types of polyps

	Type of polyp		Number: Mutated samples (%)	Mutated codon(s)
Neoplastic polyps	Adenomatosis polyps	TA	3:15(27.3)	242,361,435, c.482+66
		TVA	1:6(9.1)	264,435
		VA	1:3(9.1)	242
	Serrated polyps		2:2(18.2)	361,386, 399, 435, c.483-4
	Juvenile polyps		0:3 (0)	0
Non-neoplastic polyps	Hyperplastic polyps		4:8 (36.4)	386, 399,399, 435
	Inflammatory polyps		0:2(0)	

**Table 6:** Comparison of mutation frequency between polyp and cancer samples

Group	Codon	Mutation	Neoplastic polyps*	Non-neoplastic*	Cancer*
1	Codon 271	AGT>AGC	0	0	2
	Codon 465	GTG>ATG	0	0	1
	C.262+80	TGT > G	0	0	2
2	Codon 242	TCA>TGA	2	0	0
	Codon 361	CGC>CAC	1	0	0
	C.482+66	CAG > T	1	0	0
3	Codon 264	AGC>AGG	1	0	2
	C.483-4	TGT> A	1	0	1
4	Codon 399	GTC>CTC	2	2	0
5	Codon 386	GGT>GAT	1	1	1
	Codon 435	ATA>GTA	3	1	5

*Number of mutated sample

**Table 7:** The frequency of mutant cancer and polyp specimens in each section of colon and rectum

Section	Location	Cancer*	Polyp *
Colon	Ascending	0:1	2:5
	Cecum	0:1	2:5
	Descending	0:1	1:5
	Hepatic flexure	1:3	1:2
	Sigmoid	1:3	1:9
	Transverse	0:1	1:3
	Splenic flexure	0:0	1:3
	Total	2:10 (20) ^§^	9:32(28.1)^§^
Rectum	Rectum	8:20 (40)	2:7(28.6)
Total		10:30 (33.3)	11:39 (28.2)

* Number of mutant: number of sample; ^§^ Number of mutant: number of sample (%).

In polyp samples, transversion was the most frequent substitution (56.25% vs. 43.75%) while in the cancer samples, transitions were detected at a higher frequency (64.3% vs. 35.7%) (Table 4). This different distribution of mutation was not statistically significant (Fisher's exact test *p*-value equals 0.3). CG>GC transversion and TA>CG transition were the most frequent substitution in polyp and cancer samples, respectively. The frequency of CG > TA transition was low in both polyp and cancer tissues (Table 4).

**Mutations and types of polyp compared with cancerous mutations**

The frequency of mutations in each type of polyps is shown in Table 5. Hyperplastic, tubular, and serrated polyps were the most mutated samples: 36.4%, 27.3%, and 18.2% of total mutations, respectively. Moreover, all serrated polyps (2/2) and half of the hyperplastic polyps were mutated. One serrated polyp had four mutations at codons 361, 386, 399, and 435 (Table 5). 

Detected mutations can be categorized into five groups: 1. Mutations seen only in cancer tissue (codons 271,465 and C.262+80); 2.

Mutations seen only in neoplastic polyps (codons 242, 361, and C.482+66); 3. Mutations seen in cancer tissues and neoplastic polyps (codon 264 and C.483-4); 4. Mutations seen in both types of polyps (codon 399); and 5. Mutations seen in cancer tissues and both types of polyps (codons 386 and 435). The frequency of each group is shown in Table 6. 

**Mutations and locations of specimens**

As shown in Table 7, most cancer and polyp samples were obtained from the rectum and colon, respectively. In cancer samples, 80% of detected mutations occurred in the rectum, while in polyps, 81.9% of mutations were identified in the colon. In other words, in cancer samples, 40% and 20% of rectum and colon specimens, respectively, were mutated, while in polyps, the percentage of mutated specimens was not different (28.1% vs. 28.6%). The association between location and mutation is considered to be statistically significant (Fisher's exact test: two-tailed *p*-value = 0.0089).

**Mutations, age, and gender**

Age at diagnosis and gender were not statistically different between patients with and without mutation in both polyp and cancer samples.

## Discussion

Colorectal cancer accounted for about 10% of cancer cases and deaths worldwide in 2018 (25). However, the molecular mechanisms of CRC remain to be elucidated. To reveal some aspects of this matter, the current study was designed to evaluate the contribution of SMAD4 alterations in colorectal carcinogenesis.

In the present study, somatic SMAD4 mutations were found in 33.3% and 28.2% of analyzed specimens with CRC and polyp, respectively. To date, varying rates of SMAD4 mutations in CRC have been reported. Based on previous reports, 2.1%-31% of CRC samples may be mutated at the SMAD4 gene (14-23).

It has been shown that mice with SMAD4 deletion or loss of SMAD4-dependent signaling have increased susceptibility to developing colorectal polyp and cancer (36). On the other hand, alterations of SMAD4 have been associated with both metastasis (19) and a significantly poor prognosis (20).

The loss of SMAD4 function causes an increased genomic instability in epithelial tumors, blocks growth inhibition and apoptosis which are normally induced by TGF-β, and promotes inflammation through TGF-β, thereby possibly paving the way for the expansion of genetically defected cells during polyposis and tumorigenesis (37).

Considering that SMAD4 gene is located at 18q21, a region where allelic loss is very prevalent in CRC (38, 39), SMAD4 may play an important role as a tumor suppressor gene, and genetic alterations may have some role in silencing SMAD4 in (a fraction of) CRC (40). 

In accordance with previous works, the majority of mutations clustered in the MH_2_ domain in both polyp and cancer, while the MH_2_ domain represents only 41.5% of the coding sequence (41). MH_2_ residues are necessary for homodimerization and hetero-oligomerization with SMAD 2 or 3 proteins (42). Therefore, mutations in this region may cause a cessation of signal transmission through the TGF-ß pathway, which has been connected to many human diseases such as cancer (43).

In agreement with previous reports on CRC (19, 22, 41), missense mutations appeared to occur more frequently. Missense mutations are presumably selected for/during tumorigenesis due to their structural impacts on a specific function (44) or locking a protein in a specified state. They can also lead to drastic destabilization of the mutant protein or alter protein binding properties and its interaction network (45, 46). For example, the majority of missense mutations outside of codons 330–370 inactivate SMAD4 through protein degradation (47). However, the extent to which cancer mutations might affect biomolecular structure and interactions remains unknown. Using structure-based methods may be helpful to predict the effects of mutations on protein stability and protein-protein interactions (48).

The rate of transition in cancer specimens was higher than transversion (64.3% vs. 35.7%). The situation was reversed in polyp samples (43.75% vs. 56.25%). The observed difference suggests that the mechanisms causing SMAD4 mutations in CRC and polyp are somewhat distinct from each other, or maybe conversion and transition of adenoma into early carcinoma needs different engines (19).

The rate of CG>TA transition was low in both polyp and cancer compared to previous reports (approximately 54%) in colorectal tumors (19). CG>TA transition is thought to result from hydrolytic deamination of 5-methylcytosine residues particularly at the CpG dinucleotide in the body of genes, outside of CpG islands. Therefore, the lower frequency of CG>TA transitions can be attributed to the global genome hypomethylation as a key initiating event in cancer development (49). The current authors’ previous work on gastritis lesions showed that global genome hypomethylation may induce a different pattern (50) and spectrum of mutations of the p53 gene in an Iranian population (51), which implies other mechanism(s) in cancer development in the Iranian population. Two of three CG>TA transitions occurred at CpG codons, i.e. codons 361 and 465; therefore, defining methylation status of these codons may be informative.

If intronic alterations occur in conserved splicing sites or introduce novel splicing sites, splicing may be somewhat affected, and therefore, protein truncation or non–functional protein may be the result. Nevertheless, if the mutation occurs in regulatory elements, it could vary gene expression. For example, the intronic mutation seen here, c.483-4, mapped in a constitutive acceptor sequence (tttctgTtag) and T > A transversion may alter the efficiency of splicing. 

The most frequently detected mutation was missense at codon 435 (Ile>Val) in CRC and polyp samples. There is no previous report about this mutation in CRC, but in Juvenile Polyposis Syndrome, it is a usual event (52). The functional consequence of two branched-chain amino acid substitution at MH_2_ domain needs in vitro and in silico evaluations. 

Changing codon 399 (Val>Leu) was the second most common mutation in polyp samples, while it was not detected in CRC samples. Therefore, this mutation is likely to have a protective effect against becoming cancerous. There are no reports of this type of mutation in CRC or polyps.

Mutations at codons 264 and 271 were the next prevalent type of alterations seen in CRC and polyp samples. These codons are located in the linker domain of SMAD4 protein, a region necessary for subcellular localization (41) and transcriptional activation through p300/CBP (53). 

Mutations of R361H were seen only in one serrated polyp, while R361H in the MH2 domain is reported as the most common mutation of the SMAD4 gene in colorectal cancer (16, 18, 19). Arg361 mapped to a conserved protein loop (L1 loop) across SMAD2, 3, 4 proteins (41). Arg 361 forms a salt bridge with Asp351 and Asp 537, which directly involves homodimerization and hetero-oligomerization with R-SMADs (54, 55). Therefore, R361H disturbs both homo- and hetero-oligomerization and is considered a pathologic mutation (41).

Mutations at codons 465, 271, and c.262+79 were detected only in CRC. Therefore, it may be concluded that these types of mutation have some advantages for later stages of colorectal carcinogenesis. Wild type codon 465 is a highly conserved residue within the MH_2_ domain of the SMAD4 protein (56); missense mutations at this codon may result in the loss of the normal function of SMAD4. Deletion at codon 465 was previously reported in CRC (57). It was further shown that missense mutations of R361 and V465 resulted in an 8% and 30% decrease in BMP signaling, respectively (58). 

Another new and important mutation (considering final outcome, not frequency) is the conversion of codon 242 (TCA) to stop codon (TGA). If left unrepaired, the nonsense mutation will eventuate in a truncated and usually nonfunctional protein. The more distant the mutant stop codon is from the original stop codon, the more decisive non-functionality is. In this case, as codon 242 locates in the middle of the linker region, the translated proteins have only a complete MH_1_ domain without an MH_2_ domain. 

This study detected some identical and several exclusive mutations in CRC and neoplastic polyps. The presence of the same *SMAD4* mutations in both CRC and neoplastic polyp (264, 386, 435, c.484-4) suggests that these mutations had occurred in the primary polyps, and then the cell population having these mutations gained the potential and permission to develop into carcinoma. Therefore, these types of mutations have specific advantages for polyposis or carcinogenesis and can be used as diagnostic or prognostic markers. On the other hand, polyp-specific mutations (242, 361, 399, c.248+64) may lower the risk of transformation of these polyps toward CRC. 

To summarize, SMAD4 alterations in CRC and polyp were investigated. The current findings showed some previously reported as well as some novel mutations. These mutations may result in the loss of multiple functional properties of SMAD4, such as communication network (homodimerization, hetero-oligomerization), subcellular localization, transcriptional activation, and altered stability compared with wild type protein, and such switching may contribute to tumorigenesis. However, their functional consequences must be evaluated. 

Due to limited access to polyp samples, especially cancerous polyps, the findings of the current study should be validated in a larger population.

## Conflict of interests

The authors declare that they have no conflict of interest.
